# NICE and Fair? Health Technology Assessment Policy Under the UK’s National Institute for Health and Care Excellence, 1999–2018

**DOI:** 10.1007/s10728-019-00381-x

**Published:** 2019-07-19

**Authors:** Victoria Charlton

**Affiliations:** Department of Global Health and Social Medicine, King’s College London, 40 Aldwych, London, WC2B 4BG UK

**Keywords:** Healthcare priority-setting, Health technology assessment, National Institute for Health and Care Excellence (NICE), Social values, Justice, Health policy, Fairness

## Abstract

The UK’s National Institute for Health and Care Excellence (NICE) is responsible for conducting health technology assessment (HTA) on behalf of the National Health Service (NHS). In seeking to justify its recommendations to the NHS about which technologies to fund, NICE claims to adopt two complementary ethical frameworks, one procedural—accountability for reasonableness (AfR)—and one substantive—an ‘ethics of opportunity costs’ (EOC) that rests primarily on the notion of allocative efficiency. This study is the first to empirically examine normative changes to NICE’s approach and to analyse whether these enhance or diminish the fairness of its decision-making, as judged against these frameworks. It finds that increasing formalisation of NICE’s approach and a weakening of the burden of proof laid on technologies undergoing HTA have together undermined its commitment to EOC. This implies a loss of allocative efficiency and a shift in the balance of how the interests of different NHS users are served, in favour of those who benefit directly from NICE’s recommendations. These changes also weaken NICE’s commitment to AfR by diminishing the publicity of its decision-making and by encouraging the adoption of rationales that cannot easily be shown to meet the relevance condition. This signals a need for either substantial reform of NICE’s approach, or more accurate communication of the ethical reasoning on which it is based. The study also highlights the need for further empirical work to explore the impact of these policy changes on NICE’s practice of HTA and to better understand how and why they have come about.

## Introduction

In any healthcare system operating with finite resources, decisions must be made about how those resources are allocated. This inevitably creates both ‘winners’ and ‘losers’, as some groups find their needs prioritised while others are prevented from accessing potentially beneficial technologies. Healthcare priority-setting is thus an essential but often contentious activity, which must be subject to robust justification if its outcomes are to be considered ethically, socially and politically acceptable.

The heated debate that often surrounds priority-setting is reflected in the experiences of the UK’s National Institute for Health and Care Excellence (NICE). Tasked by the UK government in 1999 with advising the National Health Service (NHS) on its adoption of new and existing health technologies, NICE is no stranger to controversy and has regularly had to justify both its decisions and its reasoning to politicians, patients and the public [23, 31, 73, 74, 76]. In the absence of any societal consensus on how the needs of NHS users should be prioritised, it has sought to demonstrate the fairness of its decision-making through reliance on two ethical frameworks, one procedural and one substantive.

The first of these, “accountability for reasonableness” (AfR), seeks to secure fairness through the pursuit of a fair process [16]. This is represented by the fulfilment of four conditions: (1) that both the decisions made and the grounds for reaching them are made public (‘publicity’); (2) that these grounds are ones that fair-minded people would agree are relevant in the particular context (‘relevance’); (3) that there are opportunities for challenging and revising decisions and resolving disputes (‘appeal and revision’), and (4) that measures are in place to ensure that the first three conditions are met (‘enforcement’) [16, 40]. A complementary substantive framework, termed an “ethics of opportunity costs” (EOC), further specifies AfR’s ‘relevance’ condition by stipulating that resources should be distributed with regard to allocative efficiency [67, 68]. Under EOC, technologies are judged primarily on their cost-effectiveness—that is, the amount of health they deliver per unit cost—compared with available alternatives, measured by the so-called incremental cost-effectiveness ratio^1^ (ICER). Through the process of health technology assessment (HTA), an individual technology’s ICER is compared against NICE’s overall cost-effectiveness threshold to indicate whether it represents an efficient use of NHS resources. This threshold theoretically represents the point at which the health benefits displaced to fund a technology (the ‘opportunity cost’) exceed the health benefits that it can be expected to deliver. Maximising efficiency would therefore require the NHS to only adopt technologies whose ICERs fall below this threshold. However, under EOC wider equity concerns are also incorporated through the deliberations of an independent appraisal committee, which make allowances for other potentially relevant social and ethical values. Relatively cost-ineffective technologies may thus be recommended if the committee judges these wider considerations important enough to justify the associated loss of allocative efficiency.

Although this approach seems ethically sound [67], the controversial nature of its work makes NICE subject to significant social, political and financial pressures [74]. These, in turn, make its methods vulnerable to adaptation in ways that might curtail or undermine its fundamental commitment to fairness. A review of the literature (“Appendix 1”) indicates that, to date, there has been no systematic study of how NICE’s approach to priority-setting has changed normatively over time and therefore no basis from which to consider the wider implications of these changes. This paper attempts to address this gap by empirically examining normative developments to NICE’s approach and analysing whether these enhance or diminish fairness, as defined by NICE’s stated reliance on the AfR and EOC ethical frameworks.

## Methods

The study adopted a mixed-methods design, comprising thematic analysis of NICE HTA policy documents and semi-structured qualitative interviews.

NICE currently operates several HTA workstreams, including the ‘core’ technology appraisal (TA) programme, established in 1999, and a dedicated Highly Specialised Technologies (HST) programme, established in 2013. This study focused on these programmes because: (1) TA is the largest and most long-established of NICE’s HTA workstreams, (2) both TA and HST are used primarily to evaluate pharmaceuticals rather than other technology types, and (3) they are unique across NICE’s workstreams in carrying a funding mandate—that is, NHS England is obliged to make funds available for the technologies that they recommend.

Thematic analysis covered 32 documents published between 1999 and 2018, including each edition of the core technical manuals relating to TA and HST. (See “Appendix 2” for a full list.) The study’s analytical approach broadly followed that described by Braun and Clarke [4]. Following familiarisation with each document, a single coder deduced an initial set of codes relating to potentially normative content.^2^ (“Appendix 3”.) Qualitative data relevant to each code were then systematically extracted and collated, with documents analysed by chronological age and further codes added inductively as required. Once collated and assigned to a code, data were analysed and mapped to identify potential themes and patterns. This process was iterated and themes refined before being used to develop an interview topic guide.

Following thematic mapping, between June and October 2017 eight semi-structured interviews were conducted with longstanding participants in (or close observers of) NICE HTA. Their purpose was to contextualise, scrutinise, validate and challenge empirical findings and their preliminary interpretation by the researcher. Each interview lasted between one and 2 hours. Interviewees were selected purposively and were intended to represent a variety of perspectives. They included an appraisal committee Chair, a NICE Board member, two senior members of NICE staff, two technical advisors, an industry representative and an academic commentator.^3^ Interviews were based on a detailed topic guide which asked interviewees to independently identify key changes to NICE’s approach before commenting on emerging findings highlighted by the researcher and possible interpretations. (“Appendix 4”.) Interviews were digitally recorded and key data (including direct quotes) extracted in note form within 48 h. Recordings were consulted as required during preparation of this paper.

## Results

Two main themes emerged from the study: (1) the formalisation of NICE’s approach to HTA, and (2) increased leniency in its evidential requirements and handling of evidence.

Before considering these themes, it should be acknowledged that the analysis also revealed a high degree of continuity in NICE’s approach and a long-standing commitment to the AfR and EOC frameworks. NICE’s use of ICERs, quality-adjusted life-years (QALYs) and the cost-effectiveness threshold as its preferred tools for decision-making, with some allowance for relevant social and ethical values, has been consistent since the institute’s inception. Linked to this, the distinction between *assessment*, in which evidence relating to a technology is collated and evaluated in order to establish an estimated ICER, and *appraisal*, in which an independent committee balances this information against other potentially relevant considerations before making a recommendation, has consistently formed the backbone of NICE HTA. Nevertheless, aspects of NICE’s approach have undergone significant change over time, with important normative implications.

## Formalisation of NICE’s Approach to HTA

Since the TA programme was established in 1999, NICE’s documented approach has become increasingly standardised, specified and detailed: that is, it has become more formalised [78].

**Fig. 1:**
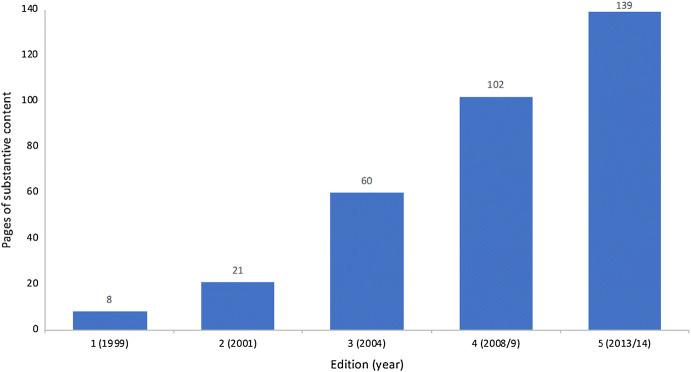
Substantive length of NICE TA technical manuals, editions 1–5 [33, 34, 36, 37, 41, 42, 46, 48]

This trend can be crudely illustrated by comparing the length of each edition of the TA technical manuals (Fig. 1). Unsurprisingly given that NICE was in the global vanguard of nationalised healthcare priority-setting, early editions of these process and methods manuals had little precedent to draw on and were thus high-level and brief. The first (combined) edition contained only eight pages of substantive content^4^ and was limited to general advice, for example, on the topics that manufacturers should “give consideration to” when submitting evidence to NICE, leaving the “format”, “length” and detailed content of such submissions unspecified [33]. As both NICE and the HTA field have matured, the level of detail that these manuals contain has increased substantially, as has their length. The current process and methods manuals contain 79- and 60-pages of substantive content respectively and are supported by a 36-page evidence submission template and a 54-page user guide, demonstrating the more demanding formal requirements that participants in NICE TA are now expected to comply with [46, 48, 49].

This increase in the volume of guidance relates to both the methods used to conduct HTA and the processes followed in applying those methods. It also spans two distinct phases of activity: assessment and appraisal.

### Formalisation of Assessment

NICE defines assessment as the systematic evaluation of evidence relating to a technology’s clinical- and cost-effectiveness [46]. It is carried out by an independent academic group, drawing heavily on a detailed submission prepared by the technology’s manufacturer, and typically results in the calculation of a technology’s estimated ICER. This precedes and provides the starting point for appraisal, during which other sources of evidence, such as expert testimony and public consultation responses, are considered alongside the wider social and ethical implications of a technology’s potential adoption. Compared with appraisal, assessment is thus a relatively technical process, in which normative considerations play a less central role. Nevertheless, its conduct has important implications for fairness.

The trend towards formalisation of assessment has been underway since 1999 but is punctuated by the introduction of the so-called ‘reference case’ in 2004: a ‘blueprint’ standardising the calculation of a technology’s ICER by limiting variation across several technical domains.^5^ Although earlier manuals had indicated a preference across several of these domains, NICE at the time stated that the manuals should be treated as “an aid to thought […] rather than as a substitute for it”, acknowledging that its preferred methods “would require interpretation in the context of each specific technology” [34]. In contrast, the norm established by the reference case can only be deviated from by exception, with ICERs derived from non-reference case analyses having to be “justified and clearly distinguished” from this norm [36]. Within the reference case, certain elements have also become more specified over time. The EuroQol-5-dimension (EQ-5D) instrument, for example, one of several tools available to measure health gain, has gradually evolved from being something that should be “ideally” used [36], to being the “preferred measure” [41], to being an instrument that would require “empirical evidence” to support its non-use [46]. Normative judgements implicit in these choices—for example, the lack of parity between physical and mental health that some argue is inherent to the EQ-5D [71]—have thus become embedded.

Another normative judgement that has become increasingly formalised is the scope of effects that can be included in the calculation of a technology’s ICER. The 1999 edition of the manual was both non-specific and inclusive: calculations could take account of a technology’s “direct and indirect costs” to the NHS, as well as unspecified “wider costs […] and benefits”, which could be presented to, and considered by, an appraisal committee [33]. Over time, more precise advice has led to the exclusion of many of these wider effects. For instance, consideration of a technology’s impact on economic productivity, expressly permitted in 1999 [33], was restricted to cases in which this was “thought to be an important element of the benefits” in 2001 [34], was “not normally” permitted by 2004 and is now explicitly “excluded” [41, 46]. Similarly, while consideration of “significant resource costs imposed outside the NHS” was permitted until 2004 [36], this is now allowed only in “exceptional circumstances” and with the prior agreement of the government [41, 46]. Thus, over time several value judgements that were previously open to deliberation have become embedded through the formalisation of assessment and closed off to committee consideration.

### Formalisation of Appraisal

Formalisation has also occurred across appraisal: the process through which an independent committee balances the information contained within the assessment report against a range of other considerations in judging whether a particular technology should be adopted by the NHS [46]. This evolution in policy has occurred in two main stages.

#### 2002–2008: Guidance Based on General Principles Developed Through Public Participation

In the early years of NICE’s existence, appraisal committees were guided in their decision-making by a set of high-level ethical principles formulated with the aid of public participation. In 2002, recognising the importance of normative judgements and acknowledging that NICE and its committees “have no particular legitimacy to determine the social values of those served by the NHS”, NICE established its Citizens Council (CC) [1, 65]. Selected as a representative sample of the UK public, the CC was periodically consulted on specific normative questions and helped NICE to develop a set of general ethical principles to guide its conduct of HTA. These were compiled in 2005 as *Social value judgements: principles for the development of NICE guidance* (SVJ), a formal policy document issued by NICE’s Board of Directors and intended for use by both internal and external stakeholders.

The first edition of SVJ contained 13 principles that endorsed or discouraged various value judgements, while acknowledging that “there will be circumstances when—for valid reasons—departures from these general principles are appropriate” [38]. The second edition, published in 2008, took a less permissive tone; it advised committees that “all NICE guidance […] should be in line with […] the social value principles set out in this document”, discouraging departure from the principles, even in exceptional circumstances [40]. However, while compliance was made more stringent, the principles themselves remained (and in some cases became more) accommodating, allowing significant room for interpretation.^6^ Thus, while SVJ was taken seriously as a source of ethical guidance, committees retained considerable freedom to exercise judgement on a case-by-case basis.

#### 2009 Onwards: Guidance Based on Decision-Rules of Unclear Provenance

Until the late 2000s, SVJ—informed by the deliberations of the CC—provided the basis for NICE’s normative approach. However, in recent years the importance of both SVJ and the CC has waned. Unlike the regularly reviewed technical manuals, SVJ has not been updated since 2008 and several interviewees suggested that it is no longer regularly consulted by appraisal committees.^7^ The CC, which used to meet once or twice a year, has not met since 2015. In place of the principle-based approach facilitated by these tools, a more rigid one based on formalised ‘decision-rules’ appears to have emerged. Much clearer expectations have therefore been set about the value judgements that committees should make and the circumstances in which they should make them.

##### Decision-Rule 1: The Cost-Effectiveness Threshold

The first decision-rule to emerge was the use of an explicit cost-effectiveness threshold, together with the specification of several value-based factors for justifying committees’ violation of this basic rule.

Under EOC, NICE’s foremost distributive concern is to support “an NHS objective of maximising health gain from limited resources” by basing its recommendations primarily on allocative efficiency: that is, cost-effectiveness [46]. Prior to 2004, this rested on appraisal committees’ “judgement” in deciding whether “on balance, the technology can be recommended as a cost-effective use of NHS resources”, with committees themselves left to identify and weigh any “significant matters of equity which might compensate” for poor cost-effectiveness [35]. In 2004, this flexible approach was replaced with more restrictive advice that a technology can normally only be deemed cost-effective if its ICER falls below or within a range of £20,000–£30,000/QALY [36]. NICE has always maintained that this ‘threshold’ is for guidance purposes only and that appraisal committees do not use “a precise maximum acceptable ICER above which a technology would automatically be defined as not cost-effective, or below which it would” [46]. However, alongside this figure the 2004 manual goes on to specify four “factors” that a committee should “make explicit reference to” if it is to recommend a technology whose ICER exceeds £20,000/QALY (Table 1) [36]. Above £30,000/QALY, “the case for supporting the technology on these factors has to be increasingly strong”, suggesting that committees should not recommend such technologies on other value-based grounds [36].

**Table 1:** Factors justifying recommendation of a technology above £20,000/QALY

2004 Methods guide (3rd edition) [36]	2008 Methods guide (4th edition) [41]	2013 Methods guide (5th edition) [46]	2008 SVJ (2nd edition) [40]
The degree of uncertainty surrounding the calculation of ICERs	The degree of certainty around the ICER. In particular, the Committee will be more cautious about recommending a technology when they are less certain about the ICERs presented	As per 2008 Methods guide	NICE should not recommend an intervention (that is, a treatment, procedure, action or programme) if there is no evidence, or not enough evidence, on which to make a clear decision
The innovative nature of the technology	The innovative nature of the technology, specifically if the innovation adds demonstrable and distinctive benefits of a substantial nature which may not have been adequately captured in the QALY measure	As per 2008 Methods guide	Decisions about whether to recommend interventions should not be based on evidence of their relative costs and benefits alone. NICE must consider other factors when developing its guidance, including the need to distribute health resources in the fairest way within society as a whole NICE can recommend that use of an intervention is restricted to a particular group of people within the population (for example, people under or over a certain age, or women only), but only in certain circumstances When choosing guidance topics, developing guidance and supporting those who put its guidance into practice, the Institute should actively consider reducing health inequalities including those associated with sex, age, race, disability and socioeconomic status
The particular features of the condition and population receiving the technology	Not included	The technology meets the criteria for special consideration as a ‘life-extending treatment at the end of life’	
Where appropriate, wider societal costs and benefits	Not included	Aspects that relate to non-health objectives of the NHS	
Not included	Whether there are strong reasons to indicate that the assessment of the change in health-related quality-of-life has been inadequately captured, and may therefore misrepresent the health utility gained	As per 2008 Methods guide	No close equivalent

Since 2004, the £20,000–£30,000/QALY threshold range has remained unchanged. However, the factors used to justify breaching it have undergone substantial revision, with a trend towards greater specification (Table 1). In particular, one initially ambiguous factor—the “particular features of the condition and population receiving the technology”—has been replaced by a highly specified decision-rule.

##### Decision-Rule 2: The ‘End of Life Rules’

Following NICE’s high-profile rejection of several late-stage cancer drugs in 2008—and the public and political protest that ensued [9, 76]—NICE’s methods were amended to give special priority to life-extending treatments for terminally ill patients. The so-called ‘end-of-life’ (EOL) rules, introduced in 2009 and updated in 2016, replace the general advice that committees consider “the particular features” of the population being treated, with a specific instruction to consider “giving greater weight to QALYs achieved in the later stages of terminal diseases” [43]. This has had the effect of increasing the cost-effectiveness threshold for these technologies (exclusively, to date, cancer drugs) to £50,000/QALY.^8^ To be evaluated under this more favourable threshold a technology is required to meet two substantive criteria: it should (1) be indicated “for patients with a short life-expectancy, normally less than 24 months”, and (2) offer “an extension to life, normally of at least an additional 3 months compared to current NHS treatment” [50]. A third criterion, that a technology be “licenced or otherwise indicated for small patient populations” was rescinded in 2016 [43]. NICE has never offered any empirical or theoretical basis for these three criteria.

##### Decision-Rule 3: QALY Weighting for Highly Specialised Technologies

A further example of formal prioritisation of a particular patient group relates to the HST programme, dedicated to drugs for very rare, usually serious conditions.

Given the small commercial market and high price often demanded for highly specialised technologies [20], the likelihood of their being recommended based on cost-effectiveness alone is very low. As such, when the HST programme was introduced in 2013 its methodology eschewed use of an explicit cost-effectiveness threshold, leaving the appraisal committee to reach a deliberative judgement that balanced “value for money” against other value-based considerations [47]. However, in 2017 this approach was replaced by a more formulaic one which stipulates both a precise cost-effectiveness threshold (£100,000/QALY) and a single value-based justification for exceeding it. According to this approach, above £100,000/QALY a committee’s “judgements” about a drug’s “acceptability” as an effective use of NHS resources “must take account of the magnitude of the incremental therapeutic improvement, as revealed through the number of additional QALY’s gained” over a patient’s lifetime [47]. More specifically, the weight assigned to each additional QALY is defined by a numerical scale, ranging from one (for technologies delivering up to ten additional QALYs) to three (for technologies delivering 30 or more additional QALYs). The HST manual does not provide any basis for this decision-rule and its use appears to conflict with SVJ’s advice that committees should “evaluate drugs to treat rare conditions […] in the same way as any other treatment” [40].

##### Decision-Rule 4: Differential Discounting of Costs and Benefits

One effect of weighting QALYs based on lifetime therapeutic improvement is to favour young patients suffering from severe or life-shortening diseases, who can potentially accrue large benefits over a long time-period. This group has also been formally prioritised through a change to NICE’s policy on discounting; the custom of valuing effects that are experienced today more highly than those that are experienced in the future [5, 12, 26].

Prior to 2011, NICE’s policy was to discount both the costs and benefits associated with a technology’s adoption at a rate of 3.5%, as recommended by the UK Treasury [41]. The normative implications of this seemingly technical judgement became apparent following the appraisal of a potentially life-saving drug for osteosarcoma—a rare childhood cancer—in which significant discounting of the long-term benefits to the children that it saved was partly responsible for the drug exceeding the usual cost-effectiveness threshold [44]. Following public criticism of the appraisal committee’s provisional decision not to recommend the drug [28, 32, 60], NICE amended its discounting policy to allow health benefits to be discounted at a lower rate of 1.5% in “special circumstances”, namely when “treatment effects are both substantial in restoring health and sustained over a very long period (normally at least 30 years)” [45]. The CC was consulted on this matter several months later and gave qualified support to NICE’s use of differential discount rates. However, it stated that it was “reluctant to compile a definitive list” of those factors that might be “relevant” in deciding when to apply this policy [55].

## Increased Leniency in Evidential Requirements and the Handling of Evidence

The second major theme emerging from this study relates to NICE’s approach to the use and handling of evidence.

### Evidential Requirements

The study identified three key examples of policy developments that have led to a weakening of the evidential requirements for recommending individual technologies.

#### Increased Willingness to Rely on Non-randomised and Indirect Study Designs

When assessing a technology’s impact, NICE has historically advocated the ‘hierarchy of evidence’ first promulgated by the evidence-based medicine movement in the early 1990s [27, 69]. This claims that “different types of study design can […] be ranked according to a hierarchy that describes their relative validity” for estimating clinical effectiveness, with randomised controlled trials (RCTs) “ranked first” compared with other study designs [36]. Thus, NICE’s manuals have historically expressed “a strong preference for evidence from ‘head-to-head’ RCTs”, with evidence derived from other study types ideally acting only to supplement this preferred source [36]. This preference is retained today, but both documentary and interview evidence suggest that NICE has become more willing to base decisions on less preferred sources of evidence. For example, recent editions of the methods manual contain substantial new material on the use of both “non-randomised and non-controlled evidence”, such as observational studies, and “indirect comparisons and network meta-analyses”—advanced statistical techniques for comparing technologies that have not been subject to a direct head-to-head trial [46]. The manual acknowledges that these methods carry “additional uncertainty”, but implies that they nevertheless provide an acceptable basis for decision-making; committees are simply advised to take this uncertainty “into account” when estimates of clinical effectiveness are “derived from indirect sources only” [46]. Interview evidence supported this interpretation, with one interviewee stating that committees today were expected to make decisions on the basis of “much much worse evidence” than in the past.

#### Subgroup Analysis

An increased tolerance of uncertainty is also apparent in NICE’s changing approach to the use of subgroups.

The consideration of how ICERs might vary across different groups of patients—for example, based on disease stage, or risk factors—has always featured in NICE’s approach. However, early editions of the manual stipulated stringent criteria for the use of subgroup analysis because of the loss of statistical power that comes from dividing a trial population into smaller subsets, and the potential for bias to be introduced through ‘data dredging’ (i.e. searching for patterns in data that have arisen from chance) [29, 70, 79]. Over time, NICE has become more permissive about the use of such analyses, in relation to both the rationales that underlie subgroup identification and the point at which this identification is made. According to NICE’s early advice, the decision to conduct subgroup analysis should be actively “justified” through “a sound biological a priori rationale” for the existence of subgroups and “evidence that clinical-effectiveness or cost-effectiveness may vary between such groups” [34]. The “credibility” of subgroup analysis would be “improved” if “confined to the primary outcome and to a few predefined subgroups”, minimising the opportunity for “ad hoc data dredging”, which was explicitly prohibited [34, 36]. Today, this requirement for “clear clinical justification” [36] has been rescinded and replaced with a far more wide-ranging basis for subgroup identification: namely “known, biologically plausible mechanisms, social characteristics or other clearly justified factors” [46]. Similarly, while “post hoc data ‘dredging’ in search of subgroup effects” is “to be avoided” and will be “viewed sceptically”, the requirement for subgroups to be identified prior to appraisal on the basis of an a priori expectation of difference has been significantly weakened; the identification of subgroups post hoc, “during the deliberations of the Appraisal Committee”, is now expressly permitted [46]. Subgroup analysis is also now performed as standard—“as part of the reference-case analysis” [46]—rather than by exception, despite the inherent uncertainty underlying such analyses.

#### Special Evidential Standards for Cancer Drugs

The most explicit change to NICE’s evidential requirements concerns technologies eligible for consideration under NICE’s EOL rules; that is, cancer drugs.

Since 2004, the methods manual has advised committees to take uncertainty into account when evaluating technologies with high ICERs, with committees discouraged from recommending adoption of technologies that appear to be both poorly cost-effective and subject to considerable uncertainty. Specifically, above ICERs of £20,000/QALY “the committee will be more cautious about recommending a technology when they are less certain about the ICERs presented” [41, 46]. This policy is in tension with the EOL rules because the drugs typically evaluated under these rules almost always have ICERs exceeding £20,000/QALY—otherwise reference to the rules would be unnecessary. They are often also subject to what one interviewee described as “huge uncertainty”, stemming from limitations in the clinical trials supporting the use of many cancer drugs, which are often of limited duration, reliant on surrogate end-points and lacking a comparator arm, as well as being industry-funded [2, 14, 17, 62]. Thus, rather than supporting the exercise of increased caution, the EOL rules provide a means for appraisal committees to recommend high-ICER technologies about whose clinical- and cost-effectiveness there remains substantial uncertainty.

Following review and revision of the EOL rules in 2016, the level of uncertainty deemed acceptable was further increased through a caveat stating that technologies assessed under the rules must merely have “the prospect of” offering a 3-month extension to life [43, 50].^9^ For technologies failing to meet this requirement, the appraisal committee has the further option of recommending their use through the Cancer Drugs Fund (CDF)—a source of ring-fenced funding in England—as long as they “display *plausible potential* for satisfying criteria for routine use” [emphasis added] (i.e. an ICER not exceeding £50,000/QALY). Thus, over time NICE’s formal policy concerning high-ICER drugs has shifted from one that suggests increased exercise of caution in cases of uncertainty, to one that actively encourages their recommendation despite such uncertainty, as long as they are indicated for cancer.

### Collation, Review and Evaluation of Evidence

Alongside changes to evidentiary requirements have been changes to the ways in which evidence is amassed and handled prior to use by an appraisal committee. This has occurred primarily through the introduction of new process variations which seek to increase the speed and efficiency of NICE HTA and reduce costs by making assessment ‘lighter touch’ and transferring workload from independent academic groups, commissioned by NICE, to industry. (Table 2).

**Table 2:** Key features of the multiple technology appraisal (MTA), single technology appraisal (STA) and fast-track appraisal (FTA) processes

	Multiple technology appraisal (MTA) [42]	Single technology appraisal (STA) [39]	Fast track appraisal (FTA) [51]
Introduction	1999	2006	2017
Scope	Single or multiple technologies in one or more indications	Single technology in a single indication; usually new drugs or new indications of existing drugs	Single technologies where the ICER is expected to be < £10,000/QALY
Responsibility for assessment	Independent assessment group (AG)	Independent evidence review group (ERG)	ERG/NICE secretariat
Scope of assessment	AG produces an estimate of clinical and cost effectiveness, based on the manufacturer’s submission and a systematic review of the literature	ERG reviews manufacturer’s submission and prepares additional analysis as required	ERG/NICE prepares a commentary and technical judgements on the manufacturer’s submission
Output	Assessment report	ERG report	Joint technical briefing
Timeline	51 weeks	43 weeks	32 weeks

Prior to 2006, all technologies assessed within NICE’s TA programme underwent multiple technology appraisal (MTA). Under MTA, responsibility for assessment rests with an independent academic group known as the “assessment group” (AG), which is responsible for preparing a comprehensive report on the clinical- and cost-effectiveness of one or more technologies (or indications) for use by the appraisal committee. Although drawing on evidence submitted directly by the manufacturer, the assessment report “provides a systematic review of the literature” and is therefore “an independent synthesis” of all of the available evidence [37]. Notably, the AG does not have any vested interest in the result of the assessment.

In 2006 a new process of single technology appraisal (STA) was introduced. Under STA, responsibility for assessment continues to rest with an independent academic group, but the remit of the renamed “evidence review group” (ERG) is limited to performing “a technical review of the manufacturer/sponsor’s evidence submission” [39]. Although the ERG may “identify gaps in the evidence base”, no independent systematic review is performed, meaning that primary responsibility for evidence collation rests with the manufacturer [39]. This trend towards increased reliance on evidence and analysis sourced from industry has continued with the introduction of fast-track appraisal (FTA) in 2017. Designed to “make available, more quickly, those technologies that NICE can be confident would fall below £10,000 per QALY”, FTA limits the role of the ERG to one of providing a “commentary” and “technical judgements” on evidence received from the manufacturer [51, 57]. It is also the only process to directly involve NICE staff in assessment, with members of the NICE secretariat working alongside the ERG to prepare a “joint technical briefing” for the appraisal committee’s use. As well as “summarising” the available evidence, this briefing sets out “the scope of potential recommendations” based on an “application of NICE’s structured decision making framework” [51]. This implies that provisional recommendations may be made within the joint briefing and in advance of committee deliberations, potentially reducing the role of the committee to one of approving decisions that have been presumed since the appraisal’s initiation (i.e. that the technology will be found to be cost-effective and recommended) and ratified by NICE staff and the ERG during assessment.

Interviewees suggested that the introduction of STA—and now FTA—reflects a shift in NICE’s onus on conducting “the most detailed, independent academic assessment that could be done” to completing appraisals quickly and efficiently, in part by relying more heavily on industry involvement. In the words of one interviewee, the introduction of STA “was the start of a slippery slope and we’ve just gone further and further down that route”.

## Discussion

This study, the first systematic examination of how NICE HTA has developed normatively over time, has identified two key themes: (1) formalisation of NICE’s approach across both assessment and appraisal, and (2) increased leniency in its evidential requirements and handling of evidence. Overall, the effect of these trends has been to undermine NICE’s commitment to both the AfR and EOC frameworks and thus the fairness of its decision-making as defined by these frameworks. The wider political, social, organisational and economic implications of these developments are beyond the scope of this paper.

### Ethical Implications of the Formalisation of HTA

A key principle of justice—implicit in both the AfR and EOC frameworks—is that of formal equality: the notion that cases that are alike in normatively relevant respects should be treated as like [11, 24]. AfR requires that decisions be made on grounds that fair-minded people would agree are relevant to the particular context. Decisions that violate formal equality by treating like groups differently therefore violate this condition, as no fair-minded person would support such grounds [66]. EOC similarly rests on formal equality in requiring that, all else being equal, technologies that are similarly cost-effective be treated similarly [67]. Given this implied need for formal equality under NICE’s stated ethical approach, formalisation has the potential to enhance fairness if it acts to increase the consistency of decision-making across similar cases. However, formal equality also requires sensitivity to context; that is, it requires that cases that are *unlike* in normatively relevant respects be treated as unlike. This sensitivity is secured under EOC by requiring that potentially relevant differences between cases be given due consideration by an appraisal committee before a decision is reached. Formalisation makes this less likely if it restricts committees in their ability to take context into account and exercise judgement on a case-by-case basis. Thus, in seeking to secure fairness through formalisation, the benefits of consistency must be balanced against the harms of insensitivity.

These benefits and harms differ across the assessment and appraisal processes. While appraisal is clearly heavily value-laden, assessment can be viewed as a more technical activity in which sensitivity to normative concerns is comparatively less important. Increased consistency through formalisation therefore may have greater potential to enhance fairness when applied to assessment than to appraisal. As one interviewee explained, NICE’s “more prescriptive” approach to assessment has led to manufacturers preparing “more standardised, better quality submissions”, making it easier for committees to compare technologies consistently, and reducing the likelihood of them misunderstanding key findings or being deliberately misled by complex technical strategies intended to flatter the technology being appraised. Of course, this is not to claim that assessment is value-free or that the formalisation of value judgements within it is unproblematic; increased consistency still comes at a price. One might argue, for example, that individual economic productivity is a relevant consideration in some contexts but not in others and that consistently ignoring it allows unlike cases to be treated as like. However, NICE could reasonably argue that this is a point on which fair-minded people disagree, meaning that the grounds for this decision remain relevant, and that any loss of sensitivity is justified by the benefits of increased consistency.

Formalising some relatively uncontentious value judgements through assessment, then, may be a sensible strategy for providing appraisal committees with a consistent starting point from which to weigh a technology’s ICER against other value-based considerations. However, formalisation of appraisal raises more significant concerns, particularly when this takes the form of highly specified decision-rules which limit committees’ potential to exercise judgement.

The oldest and most fundamental of these rules is the £20,000–£30,000/QALY threshold, which provides the touchstone for defining cost-effectiveness under EOC. If applied strictly, the existence of an explicit threshold reduces sensitivity to normative concerns substantially. However, as NICE makes clear, the threshold is deliberately stated as a range so as to provide committees with space to exercise judgement, and it is also formally defeasible: that is, it can be revised or overridden in appropriate circumstances [6]. Thus, while a technology’s ICER forms the starting point for discussion, the appraisal committee exists to ensure that other relevant factors are also considered and, if they are of sufficient weight, used to justify exceeding the threshold. Thus, the rule’s relative indeterminacy and defeasibility ensure a degree of consistency in decision-making while allowing committees freedom to exercise judgement and remain sensitive to relevant differences.

Over time, however, NICE’s policy concerning the threshold has become more restrictive. The manuals’ increasing specification of those circumstances in which exceptions can be made, together with the introduction of the three decision-rules highlighted above, significantly restricts committees’ freedom to exercise judgement by defining and limiting both the relevant ways in which cases can differ and the appropriate response to these differences. The EOL rules, for example, reduce a complex normative judgement concerning prioritisation of the terminally ill to positive questions about whether or not particular substantive criteria have been satisfied and a precise maximum threshold of £50,000/QALY. They are insensitive to potentially relevant differences within these criteria—for instance, a technology that offers a 3-month extension to life is treated the same as one that extends life by 5 years—and they limit committees’ freedom to consider other potentially relevant factors, such as the age of those concerned. The relevance of some of these formal criteria can also be questioned, with population studies and deliberations of NICE’s CC suggesting that fair-minded people might not support some of the values on which these rules are based [10, 30, 53, 54, 56, 72].

While committees are not explicitly prevented from exercising judgement in interpreting and applying these more specified rules, it is arguably naïve to consider them defeasible in any meaningful sense. In the politically charged, high-profile environment in which committees operate, the establishment of formal norms creates strong expectations that are difficult to disappoint, particularly when this would mean saying ‘no’ to a technology that might otherwise be recommended. Indeed, doing so might leave committees’ decisions open to appeal or even judicial review.^10^ As one interviewee put it, the existence of these rules means that “there is less scope for committee members to use their judgement” during appraisal, with the result that appraisal becomes a “rubber stamping exercise” to ratify judgements embedded in the rules, rather than an exercise in deliberative decision-making. It thereby undermines NICE’s commitment to a distributive framework that relies on deliberation to identify and respond to relevant normative considerations.

These rules also redefine what can be considered cost-effective by increasing the threshold against which technologies are judged. Assuming that the ‘true’ opportunity cost to the NHS does lie somewhere between £20,000/QALY and £30,000/QALY, such ‘threshold creep’ implies a significant loss of efficiency, reducing the total amount of health the NHS can deliver. This could be justified under both AfR and EOC if the value judgements precipitating it could be shown to be relevant and arrived at through case-by-case deliberation. However, as described above, this cannot be demonstrated. As such, the formalisation of appraisal through the introduction of decision-rules that seek to define normatively relevant considerations and guide committees’ response to them can be shown to undermine both AfR and EOC.

Formalisation also poses a challenge to AfR’s publicity condition. This requires that the rationales on which decisions are based are publicly available, but value judgements that are embedded in the assessment process cannot be considered truly ‘public’. The policy to consistently exclude economic productivity from the calculation of a technology’s ICER, for example, is currently made 45-pages into a 95-page technical manual and is characterised as a technical requirement rather than a value judgement [46]. It also goes unmentioned in SVJ, which stakeholders might reasonably expect to provide an overview of the rationales adopted as standard in NICE’s approach, and is unlikely to form a subject of deliberation during public appraisal committee meetings because the decision to exclude it as a consideration has already been made [40]. Value judgements based on complex quantitative criteria (e.g. the EOL rules) similarly suffer from a potential loss of publicity because their application centres on whether or not a given technology satisfies the necessary substantive criteria; largely a matter for technical discussion during the relatively closed process of assessment, rather than deliberation during the more open process of appraisal. A counter argument, of course, is that less formal, more discretionary modes of decision-making pose even greater challenges to publicity because the complex rationales used may be difficult to fully convey and record on paper; further research is needed to establish whether this is the case for the relatively detailed documentation surrounding each NICE technology appraisal. Nevertheless, the lack of transparency of value judgements embedded within processes that appear exclusively technical remains, and is arguably made more insidious by the expectation—not present under a purely discretionary approach—that formal norms will be clearly stated and fully adhered to.

A further challenge to both the relevance and publicity conditions stems from the arbitrariness of the criteria on which these decision-rules are based, none of which have been publicly justified on either empirical or theoretical grounds. While rigorous academic arguments would not necessarily be expected from a policy body such as NICE, failure to provide any justification for such criteria makes NICE vulnerable to accusations that they are based on political expediency rather than morally relevant considerations. For example, several interviewees suggested that the £50,000/QALY threshold implied by the EOL rules was initially based on the political need to approve a particular cancer drug whose ICER was close to this figure, and that the change in discounting policy was similarly precipitated by the desire to recommend a particular drug [9, 59, 61, 63]. As well as undermining the relevance condition, failure to publicly justify these changes—or to acknowledge the political motivations underlying them—weakens or even contravenes AfR’s requirement that the grounds for reaching priority-setting decisions are made public.

### Ethical Implications of NICE’s Changing Approach to Evidence

This study also demonstrates several ways in which NICE’s changing approach to the use and handling of evidence has led to a weakening of the burden of proof for recommending a technology’s adoption. This has important implications for NICE’s commitment to AfR and, in particular, the allocative efficiency so highy valued through EOC.

The study has highlighted three ways in which NICE’s use of evidence has changed over time: through (1) an increased willingness to rely on non-randomised and indirect study designs; (2) more frequent and generalised use of subgroup analysis, and (3) the lowering of evidential requirements in relation to cancer drugs. In the case of both (1) and (2), the implications for NICE’s tolerance of uncertainty is, in principle, mixed. The traditional hierarchy of evidence of which RCTs are the pinnacle has been subject to much criticism [3, 7, 64, 80, 81] and NICE’s formal move away from the hierarchy does not require that committees base their decisions on poor evidence. However, as several interviewees pointed out, political and organisational pressures, as well as pragmatic considerations, make it difficult for committees to ‘shirk’ decision-making on the basis of insufficient evidence, particularly when NICE’s formal policy signals a willingness to rely on study types that are inherently subject to high levels of uncertainty and when there is little prospect of more, or ‘better’, evidence becoming available in the future. The implications of changes to NICE’s policy on subgroup analysis is similarly ambivalent in principle, but likely not in practice. In theory, subgroups can be used either ‘positively’, to identify a cost-effective group for which a technology that is cost-ineffective overall can be recommended, or ‘negatively’, to exclude a specific cost-ineffective group from a population that is cost-effective overall. In practice, interviewees made clear that the former is much easier to employ than the latter. In the words of one, if committees “think that something is going to be cost-effective on average, then they’re not looking for subgroups to say ‘no’ to”, whereas “the other way around, in my experience, they would be looking for subgroups” to say ‘yes’ to”. Thus, as another interviewee put it, subgroup analysis is primarily “a way of making things available” to patients who would otherwise not have access, despite the greater relative uncertainty associated with this type of analysis. The observed changes to policy on both study types and subgroup analysis are therefore likely to increase committees’ tolerance of uncertainty and weaken the burden of proof to which technologies undergoing HTA are subject.

In the case of cancer drugs, recent changes weaken this burden of proof to the point that it is arguably reversed. Under the revised EOL rules, cancer drugs need only demonstrate “the prospect of” offering a 3-month extension to life to warrant recommendation up to the £50,000/QALY limit, or the “plausible potential” for achieving cost-effectiveness (according to this limit) for inclusion in England’s Cancer Drugs Fund [50]. This implies that such a drug should only be rejected outright if it can be shown to have *no* prospect of offering the required extension to life, and *no* plausible potential for achieving the required ICER. This shifts the burden of proof from the manufacturer, who was previously responsible for demonstrating that their drug likely *met* the criteria for cost-effectiveness, towards the appraisal committee, which in order to reject such a drug now needs to demonstrate that it likely *does not*. Thus, the likelihood that technologies will be recommended on the basis of over-optimistic estimates of cost-effectiveness is increased.

Changes to the way in which evidence is collated, reviewed and evaluated further enhance this risk by increasing NICE’s vulnerability to bias. Following its introduction in 2006, STA quickly replaced MTA as the most utilised process and the majority of appraisals are now conducted via STA [8, 52]. However, both STA and FTA involve significantly less independent oversight than MTA and transfer responsibility for collating, presenting and analysing the evidence on which assessment is based to the party with the most to gain from a recommendation: the manufacturer. If this could be shown to lead to clearly cost-effective technologies being adopted by the NHS more quickly and at less administrative cost, then this ‘lighter touch’ approach could be justified as both good for the patient and as fair to the manufacturer. However, given the strong financial incentive that manufacturers operate under and the many sources of uncertainty inherent to HTA, transfer of responsibility for assessment to the manufacturer raises the potential for bias to permeate the many assumptions and judgements that contribute to calculation of a technology’s ICER. Of course, these judgements remain subject to independent oversight by the evidence review group (ERG). But given that the manufacturer’s ‘base case’ ICER will almost inevitably support the technology’s recommendation, under STA and FTA the ERG is tasked with effectively having to *disprove* this base case if it is to demonstrate that a technology is not cost-effective. Once more, the burden of proof has shifted away from the manufacturer. Finally, in the case of FTA there is the further risk that ERGs and appraisal committees themselves will be subject to confirmation bias—the tendency to seek evidence that might confirm or verify existing beliefs or conjectures (e.g. that a technology is cost-effective) and to ignore evidence that might disconfirm or refute them [15].

In theory, the weakening of the burden of proof that these changes together make likely could either enhance or undermine NICE’s commitment to the EOC framework. EOC requires that, equity considerations aside, technologies that are demonstrably cost-effective are adopted while those that are not are rejected. Given that HTA is invariably subject to uncertainty, there is always a risk that ‘errors’ will occur: that is, a cost-effective technology will be inaccurately classed as not cost-effective, or vice versa. To prevent such errors from occurring, it is essential, first, that the threshold against which cost-effectiveness is judged is appropriate. However, burden of proof also plays an important role. The likelihood of cost-effective technologies being incorrectly classed as not cost-effective is increased if the burden of proof is high and if it rests on the technology being appraised (i.e. if there is a presumption of cost-ineffectiveness). Conversely, if the burden of proof is reduced or reversed (i.e. if there is a presumption of cost-effectiveness), there is an increase in the likelihood of cost-ineffective technologies being wrongly classified as cost-effective. Both errors reduce allocative efficiency and—in the absence of other equity-based considerations—are therefore incompatible with fairness as defined by EOC.

The question of whether the observed reduction in NICE’s burden of proof has, in practice, led to an overall loss of efficiency is a matter for empirical research and cannot be proven here. However, there are strong reasons to suggest that it has. Empirical evidence indicates that NICE’s basic £20,000–£30,000/QALY threshold significantly overestimates the opportunity cost to the NHS, implying that many technologies are being wrongly classed as cost-effective [13]. If these findings are accurate, then the de facto increase in the threshold brought about by the formalisation of NICE’s appraisal process has likely led to further loss of NHS efficiency. Against this backdrop, additional policy developments that allow for greater tolerance of uncertainty and increased risk of bias in favour of the technology being appraised inevitably increase the likelihood that cost-ineffective technologies are being recommended, further reducing efficiency. That is, these trends are likely additive.

As a result of these changes, interviewees consistently believed that technologies that NICE would have rejected 15 years ago were regularly being recommended today, leading to a fundamental shift in how the interests of different NHS users are balanced in favour of those who stand to benefit directly from access to such technologies. Thus, the needs of the ‘average’ NHS user are deprioritised, as existing interventions and NHS services are displaced to fund the new, branded technologies that NICE typically appraises. In the words of one interviewee, “the benefit of the doubt” under current policy is “always in one direction: the patient in front of you, the recipient in front of you, the manufacturer in front of you”, with committees “almost ignoring the fact that [recommending a technology] would impose costs on others”. This shift has occurred gradually, without any public disclosure of the underlying rationale for this pattern of change, undermining NICE’s commitment to AfR’s publicity condition, and is based on formalised value judgements that are themselves open to question. If the tangible result of these changes is to reduce the efficiency of the NHS—as seems likely—then they considerably weaken the EOC framework, further undermining NICE’s commitment to publicity by contributing to an inaccurate representation of NICE’s substantive approach. As such, while certain elements of NICE’s evolving approach to HTA have the potential to enhance fairness, the overall effect of these changes has been to undermine it through a weakening of NICE’s commitment to both AfR and EOC.

## Conclusion

This empirical exploration of normative changes to NICE’s HTA approach has highlighted two concerning trends, which together appear to increase the likelihood that poorly cost-effective technologies will be recommended for adoption by the NHS. Further research is needed to understand why these changes have come about, but we might reasonably speculate that political pressures have played a part. Although legally a non-departmental (or “arms’ length”) public body and operationally independent of the Department of Health, NICE remains financially reliant on the government and has therefore had to robustly justify its continued existence during a period of significant change for the health service and the adoption of numerous austerity measures [58]. Given the government’s repeated reference to the life sciences industry as a key driver of economic growth [18, 25], and the importance of accelerating access to innovative new therapies—for the benefit of both patients and the national economy [19]—it is perhaps unsurprising that NICE’s own policies have evolved in line with these wider political aims. Global efforts to speed up the adoption of effective new treatments [21, 22, 77] may likewise have exerted pressure on NICE to recommend adoption of ‘innovative’ health technologies, as well as influencing the quantity and quality of evidence available to committees attempting to establish the cost-effectiveness of such products [17]. Finally, changes to key personnel within NICE and other stakeholders, public and patient pressure facilitated through both traditional and social media, and shifts in the wider social and political environment in which NICE operates may have each played a role in its apparent drift towards more permissive treatment of the technologies that it appraises.

Speculative as these hypotheses may be, this paper has demonstrated a policy trend that appears to undermine NICE’s proclaimed commitment to procedural and distributive justice as conceptualised through AfR and EOC. Further empirical work is required to explore the impact of these changes to policy on practice. However, it seems likely that the overall effect has been to undermine the fairness of NICE’s decision-making and reduce NHS efficiency in a manner that cannot easily be justified. A substantial *volte*-*face*—either to NICE’s methods or to the ethical frameworks on which it claims these are based—may be required if coherence is to be restored to its approach. Such widescale change may prove unfeasible in the short-term. But if NICE is to remain credible in the eyes of the public in whose interests it claims to act, there is a need for it to more accurately communicate the normative judgements on which its decisions are based, either through an update to SVJ or through an entirely new attempt to explicate its ethical approach.

## Appendix 1: Literature Review

A semi-systematic literature review was conducted between April and July 2017. The review focused on literature addressing the following question: ‘How has the process, methods and ethical framework used by NICE in its technology appraisal programme changed over time and what are the implications for NICE’s treatment of social and ethical values?’. Four databases—PubMed, Scopus, ProQuest and Web of Science—were systematically searched using search terms developed for a prior systematic literature review and a review of the terms used in pre-identified relevant papers. In addition, the Health Management Information Consortium (HMIC) and Social Policy and Practice Research (SPPR) databases were searched to identify relevant grey literature.

The search terms used across the review were as follows: Title/abstract/key (NICE OR “National Institute for Clinical Excellence” OR National Institute of Clinical Excellence” OR “National Institute for Health and Clinical Excellence” or “National Institute of Health and Clinical Excellence” OR “National Institute for Health and Care Excellence” OR “National Institute of Health and Care Excellence”) AND Title/abstract/key (“technology assessment” OR “technology appraisal” OR HTA OR “cost benefit analysis” OR “cost effectiveness” OR “comparative effectiveness research” OR “economic evaluation” OR “healthcare rationing” OR “health care rationing” OR “resource allocation” OR “health priorities” OR “healthcare priorities” OR “health care priorities” OR “priority setting” OR “health technology prioritisation” OR “health technology prioritization” OR “reimbursement decision” OR QALY OR “quality adjusted life year” OR ICER)

The review identified several papers evaluating individual changes to NICE’s methods and trends in NICE decision-making over time. However, it did not identify any empirical study of developments to NICE’s approach as a whole or of the potential ethical implications of these changes.

## Appendix 2: Documents Included in Systematic Review of NICE Policy

**Table:**

Year	Key policy documents*	Supporting documents
1999	Appraisal of new and existing technologies: interim guidance for manufacturers and sponsors	
2001	Guide to the technology appraisal process (1st ed.) Guidance for manufacturers and sponsors/Guide to the methods of technology appraisal (1st ed.)^#^ Guidance for appellants	Guidance for healthcare professional groups Guidance for patient/carer groups
2004	Guide to the methods of technology appraisal (2nd ed.) Guide to the technology appraisal process (2nd ed.) Technology appraisal process: guidance for appellants	A guide for manufacturers and sponsors A guide for healthcare professional groups A guide for NHS organisations A guide for patient/carer groups
2005	Social value judgements: principles for the development of NICE guidance (1st ed.)	
2006	Guide to the single technology appraisal process (1st ed.)	
2007		Single technology appraisal process: update
2008	Guide to the methods of technology appraisal (3rd ed.) Social value judgements: principles for the development of NICE guidance (2nd ed.)	
2009	Guide to the single technology appraisal process (2nd ed.) Guide to the multiple technology appraisal process (3rd ed.)	Supplementary advice: appraising life-extending, end-of-life treatments
2010	Appeals process guide	
2011		Clarification on discounting
2013	Guide to the methods of technology appraisal (4th ed.)	
2014	Guide to the processes of technology appraisal (4th ed.) Guide to the technology appraisal and highly specialised technologies appeal process	
2016		Addendum A—final amendments to the NICE technology appraisal processes and methods guides to support the proposed new cancer drugs fund arrangements Rapid re-consideration of drugs currently funded through the cancer drugs fund
2017		Fast track appraisal: addendum to the Guide to the processes of technology appraisal Cost comparison: addendum to the Guide to the processes of technology appraisal Procedure for varying the funding requirement to take account of net budget impact
2018	Guide to the processes of technology appraisal (4th ed.)—2018 update	

*Key policy documents include the methods guides, process guides and social value judgements documents. Versions of these guides that tailor the same information for a more specialised audience (e.g. patient/carer groups, NHS organisations), plus any amendment or addendums to these key documents, have been classed as supporting documents

#The 2001 guidance for manufacturers and sponsors document contained detailed information on the methods of technology appraisal, much of which went on to inform the development of the first formal methods guide in 2004. As such, it has been classed here as the first edition of the methods guide

## Appendix 3: Initial Codes Generated Deductively During Familiarisation Stage of Thematic Analysis

**Table:**

**#**	Code	Description
1	Fundamental principle	Any general principles identified by the document
2	Topic selection process	Process through which topics/technologies for appraisal are selected
3	Topic selection criteria	Criteria according to which topics/technologies for appraisal are selected
4	Outcomes	Health and non-health outcomes accounted for directly in the technology evaluation
5	Costs	Costs to the health service and elsewhere accounted for directly in the technology evaluation
6	Economic evaluation	Type of economic evaluation and methodology used to measure and value health effects. E.g. cost-utility analysis using QALYs based on EQ-5D
7	Acceptable evidence	The types of evidence considered acceptable in making an evaluation
8	Clinical effectiveness	Judgemental factors that can be taken into account in evaluating clinical effectiveness e.g. nature and quality of evidence, uncertainty, existing alternatives, patients views on outcomes
9	Cost effectiveness	Judgemental factors that can be considered in evaluating cost effectiveness e.g. patient perspectives on quality of life, wider societal benefit
10	Participants	Groups and individuals formally invited to participate in the appraisal process
11	Discounting policy	Discount rate(s) applied and the policy surrounding their application
12	Time horizon	Policy regarding the period of time over which costs/benefits can be calculated
13	Innovation	Any policy regarding how innovation should be treated and valued during appraisal
14	Social value judgements	Any social or ethical values explicitly regarded as relevant to appraisal and decision-making
15	Equalities considerations	Any specific considerations to be made regarding the potential for inequalities
16	Excluded considerations	Any considerations explicitly excluded from consideration during appraisal and decision-making
17	Threshold	Any explicitly stated cost-effectiveness threshold(s) or threshold range(s)
18	Resource impact	Any policy regarding the consideration of resource impact, budget impact or affordability during appraisal
19	Appeal criteria	Criteria according to which appellants may be heard

## Appendix 4: Interview Guide

Note: This basic topic guide was adjusted as appropriate between interviews.

**Table:**

Topic	Questions
Relationship with NICE	*I’ll begin by trying to understand a bit more about your relationship with NICE and the role that you played in the development of some of its key guidance documents* 1. As [role], what were your key responsibilities? What role did you play in the development of NICE’s approach to technology appraisal? *For example, how involved were you in the various updates to the Process and Methods guides?* How would you describe your current relationship with NICE? Are you involved in any ongoing work to update NICE’s *Process* guide, *Methods* guide or *Social Value Judgements* document?
View on key changes to approach	*This project is focusing on how the process guide, methods guide and SVJ document have changed over time and the implications of these changes for NICE’s treatment of social and ethical values. As such, I’m now going to ask you some quite explorative questions about how you perceive NICE’s approach to have evolved over time. Please don’t worry if you’re not able to provide detailed answers*—*a general perspective is absolutely fine* 2. Over the period of your involvement with NICE, what do you consider to have been the most important changes in its approach to technology appraisal? *By approach, I mean its process, methods or guidance on social or ethical values.* Can you identify any overarching patterns or trends in these changes? What do you think are the ethical implications of these changes?
Social value judgements document	*I’m now going to ask some questions focused on the social value judgements (or ‘SVJ’) document and its relationship with other key pieces of NICE guidance. I’ll then go on to briefly consider SVJ’s role in the practice of NICE’s technology appraisal committees* 3. In your experience, what role has the *SVJ* document played in the development of NICE’s *process* and *methods* guides? What role (if any) has the SVJ document played in the development of ad hoc amendments to these guides? *For example, the 2009 ‘End*-*of*-*life’ addendum to the Methods guide and the 2011 ‘Clarification on discounting’* Can you think of any ways in which NICE’s process and methods guides deviate from the principles set out in the corresponding versions SVJ? *I have a copy here of the eight principles set out in the most recent 2008 SVJ document if you would like to refer to them* *Moving on to consider the role of the SVJ document in the practice of technology appraisal:* Do you think the *SVJ* document has become more or less relevant to the practice of NICE’s technology appraisal committees since the first edition was issued in 2005?
Citizens Council	*I’d now like to briefly explore the changing status of NICE’s Citizens Council* 4. What role has the Citizens Council played in the development of NICE’s *Process* and *Methods* guides? How have you observed this role to have changed over time? In your view, are there any aspects of NICE’s process or methodology that conflict with advice given by the Citizens Council?
Perspective taken during assessment	*I’m now going to explore NICE’s approach to several specific social or ethical values*—*this is so that you can help me to either validate or challenge some of the hypotheses that have emerged from this project so far. As a reminder, I am defining a social or ethical value to be any factor besides clinical*- *or cost*-*effectiveness that is taken into consideration, either directly or indirectly, during technology appraisal* *NICE has changed its stance several times on whether non*-*health impacts should be taken into consideration when assessing the cost*-*effectiveness new health technologies. For example, until 2004 both productivity gains and other non*-*NHS impacts*—*such as gains experienced by other government departments*—*could be included in the calculation of cost*-*effectiveness. By contrast, under current guidance productivity is explicitly rejected as a relevant consideration and non*-*NHS impacts can only be included in exceptional circumstances, with prior agreement from the Department of Health, and cannot be included in the main ICER calculation* 5. Do you agree that NICE’s official guidance has increasingly delimited the types of costs and benefits that can be ‘counted’ during health technology assessment? *[If yes]:* What do you think are the reasons for this change? *[If yes]:* In your view, has this change in perspective also been reflected in the practice of appraisal committees? *In other words, are committees happy to take into consideration benefits presented alongside the reference case, or is the reference case analysis the main driver for decision*-*making?* Why do you think NICE has now singled out productivity as an inappropriate factor for consideration during health technology assessment?
Evidence and uncertainty	*I’d like now to briefly discuss how NICE’s approach to evidence and uncertainty has evolved.* *Historically, the Methods guide has indicated a strong preference for data derived from RCTs. However, over time this preference has been less strongly expressed and the guide has provided more detailed advice on how alternative sources of evidence might be handled; for example, data derived from indirect comparisons and modelling* 6. Does this increased focus on non-RCT data indicate a greater willingness by NICE to make decisions based on less robust evidence? *Historically, committees were advised to take the degree of certainty into account when recommending technologies at high ICERs and to exercise more caution when there is significant uncertainty about clinical or cost effectiveness. This principle still exists, but appears to be in tension with amendments brought about by the end*-*of*-*life rules and the new cancer drugs fund, the wording of which seem to allow for a relatively high level of uncertainty at ICERs significantly above the usual threshold* *Another way of looking at this is that the ‘benefit of the doubt’ has shifted, for cancer drugs at least, from the unidentified NHS patient whose interests are protected by ensuring that technologies representing poor value for money are not recommended, to the particular group of patients whose interests are served by recommending a particular technology, even if this carries an opportunity cost for the NHS* Do you agree with this hypothesis? *[If yes]:* What has been the main driver of this shift? *[If yes}:* Do you think that this shift in the benefit of the doubt applies only to cancer drugs, or has it also been adopted for other technology types? If so, which? *[If no]:* What alternative hypothesis would you put forward for the wording changes referred to above?
Innovation	*I’m now going to move on to the topic of innovation* *Ever since its creation in 1999, NICE has had a statutory responsibility to support innovation. However, the tone of the statements describing this responsibility has arguably become stronger over time. (I’ve pulled together a few examples, here, for you to glance at, if you’d like to see some of the supporting evidence for this hypothesis.)* *Several recent changes to NICE’s process have also been justified on the grounds that they accelerate access to innovation; for example, the introduction of the single technology appraisal process in 2009, which was intended to accelerate the appraisal process and enable it to be initiated closer to product launch* 7. Do you think it is fair to say that NICE has become more actively ‘pro-innovation’ in recent years? *[If yes]:* What do you think has been the main driver of this change? *[If yes]:* How do you think this change in attitude has been reflected in changes to NICE’s process and methodology? *For example, in its appraisal timelines, topic selection criteria, evidence requirements, types of recommendation?* *The current version of SVJ states that “NICE should not recommend a technology if there is no evidence, or not enough evidence, on which to make a clear decision”.* Do you think that the desire to promote innovation has sometimes led to this principle being overridden? *[If yes]:* Do you have any concrete examples for this? *The current Methods guide suggests that the innovative nature of a new health technology should only be considered at high ICERs, and only when the innovation adds “demonstrable and distinctive benefits of a substantial nature” that have not been captured in the reference case calculation* Does this accord with your experience or perception of how appraisal committees respond to innovative products in practice?
Discounting	*I’m now going to move on to the subject of discounting and how changes to NICE’s discounting policy may relate to its social or ethical value judgements* 8. What do you think were the factors driving the Supplementary guidance on discounting issued in 2011 and the subsequent change in recommended discount rates? Do you think that the reasons for these changes were purely technical or did social or ethical values also play a role? *For example, the desire to prioritise treatments targeting children and young people?*
Formalisation (appraisal)	*The case of discounting appears to be one of several instances in which social or ethical value judgements previously addressed through deliberation and the discretionary decision*-*making of appraisal committees are becoming more prescriptive and ‘rule*-*based’. Moreover, the rules often tend to introduce quantitative criteria rather than providing guidance for deliberation. Other examples include the ‘End*-*of*-*Life’ rules (which introduced several quantitative ‘cut*-*offs’), the increasingly stringent criteria concerning consideration of non*-*health benefits, and the proposed use of QALY weighting in the highly specialised technology appraisal process. [Expand on EoL example and/or mention BI test if necessary]* 9. Bearing our conversation so far in mind, do you think it is accurate to say that NICE has sought to make its social and ethical value judgements more ‘rule-based’ in recent years? Several of the recently introduced rules employ numerical ranges and cut-offs. Do you think that this reflects an emerging preference in NICE’s approach for quantitative decision-making over deliberative approaches? *Technology assessment is described by NICE as a three*-*stage process, consisting of scoping, assessment and appraisal. This sheet summarises what each stage involves, in case a refresher would be useful* Do you agree that social and ethical value judgements are increasingly being made in the scoping and assessment phases, rather than the appraisal stage? [*If yes]:* Why do you think this trend has emerged?
Formalisation (assessment)	*A review of the five editions of the Methods guide indicates that aspects of NICE’s methodology other than its social and ethical value judgments have also become more prescriptive, or ‘rule*-*based’, over time, particularly since the introduction of the reference case in 2004. A general indicator of this is the increasing length of these documents: from 12 pages in 1999, to 54 pages in 2004, to 94 pages in 2013* 10. The 2001 *Guidance for Manufacturers and Sponsors* states that it “should be seen as an aid to thought during the process of submission rather than as a substitute for it”. Do you think that this advice still applies to the current Methods guide, or has NICE’s methodology become less flexible over time? *The number of NICE appraisal programmes has increased over time, from two core programmes in the early 2000s [technology appraisal and clinical guidelines] to six today, several of which also have multiple process variations (e.g. MTA, STA, FTA)* Is it accurate to say that this expansion in the number of NICE programmes and processes has been necessitated in part by the reduced flexibility of the core technology appraisal programme? In your experience, how does NICE address technologies that cannot easily be appraised through its standard methodology?
Political landscape (relationship with government)	*I’m going to finish by asking two sets of questions concerning NICE’s role in the political landscape. These are much more explorative than the last few questions, so they could be a little challenging to answer on the spot. My first question concerns NICE’s changing relationship with government and the extent to which it is able to act independently in developing its approach to technology appraisal* 11. How would you characterise NICE’s relationship with the most recent government and how do you think this compares with its relationship with previous governments? How do you think that government priorities are reflected in the way that NICE conducts technology appraisal? *For example, in the process of topic selection? In NICE’s approach to innovation? In its consideration of wider societal impacts?* *Several of the more controversial recent changes to NICE’s approach have been issued by NICE’s Board, rather than through a full methodology review. [For example, the End*-*of*-*life rules, supplementary advice on discounting, budget impact test.]* Do you think that this reflects an increase in the political pressure NICE is now exposed to?
Political landscape (NHS)	*My last question focuses on the relationship between NICE’s methodology and the financial pressures faced by the NHS* *12. NICE’s cost*-*effectiveness threshold hasn’t formally changed since 1999, but financial pressure on the NHS has increased significantly during this period.* What techniques, if any, do you think NICE has employed over the years to maintain the overall affordability of its advice for the NHS? Would you say that the budget impact of individual technologies has become more or less relevant to decision-making as NICE’s methodology has evolved over time? *In a public health system funded by finite resources, tension inevitably arises between the needs of the individual and the needs of the population. This tension arguably becomes more acute as the system experiences greater financial stress. Principle 5 of the current SVJ document states that “although NICE accepts that individual NHS users will expect to receive treatments to which their condition will respond, this should not impose a requirement on NICE’s advisory bodies to recommend technologies that are not effective, or are not cost effective enough to provide the best value to users of the NHS as a whole”* Do you think that NICE’s methodology, overall, remains compliant with this principle?
AOB	13. Is there anything else you’d like to add or discuss that we haven’t covered?

## Footnotes

1 A technology’s ICER is calculated by dividing the incremental change in expected costs by the incremental change in expected health effects. The latter is measured in quality-adjusted life-years (QALYs). The QALY provides a quantitative measure of a technology’s effect on both quantity and quality of life, measured in life-years and on a 0–1 scale respectively.

2 Content was classed as normative if it had potential implications for NICE’s treatment of social and ethical values, defined as any evaluative consideration that does not solely contribute to the calculation of a technology’s ICER.

3 Some of these individuals were no longer in these roles at the time of interview.

4 Excludes title pages, contents, forewords and appendices.

5 These include but are not limited to: the type of economic evaluation used; the unit of measurement of health gain; the method by which such gains are measured and valued, and the discount rate applied.

6 For example, in 2005 principle 6 advised that access to technologies should only be limited to particular age groups when “there is clear evidence of differences in the clinical effectiveness” across these groups. In 2008, this principle was broadened to include “other reasons relating to fairness for society as a whole”, alongside differential clinical-effectiveness.

7 Since this work was carried out in 2017, NICE has issued a consultation on “the principles that guide the development of NICE guidance and standards”. This appears intended to supercede SVJ and states that “although much of what it [SVJ] contains is still relevant to our work, we consider that it is no longer the best way to communicate the principles”.

8 Although the original articulation of the EOL rules did not specify the magnitude of the additional weight to be applied to QALYs gained at the end-of-life, a maximum multiplier of 1.7—implying an adjusted upper limit to the threshold range of £50,000/QALY—was quickly established in practice and has since been formalised in the current methods guide.

9 The original articulation of the rules required that there be “sufficient evidence to indicate that the treatment offers an extension to life, normally of at least an additional 3 months, compared to current NHS treatment”.

10 It is feasible that a committee’s decision not to recommend a technology could be subject to judicial review on the grounds that substantive reasonable expectations established by the existence and previous operation of formal norms had not been met. The manufacturer of a technology that demonstrably met NICE’s end-of-life criteria, for example, might claim that a committee’s decision to reject it at an ICER < £50,000/QALY was unlawful on these grounds. Even if such a claim were likely to be unsuccessful, as experience seems to suggest [75], fear of judicial review might nevertheless make committees reluctant to make such a recommendation.
